# Influence of irradiation distance on the mechanical performances of resin composites polymerized with high-irradiance light curing units

**DOI:** 10.1186/s40824-022-00267-5

**Published:** 2022-05-20

**Authors:** Soram Oh, Hyun Ju Kim, Hyun-Jung Kim, Sibel A. Antonson, Sun-Young Kim

**Affiliations:** 1 Department of Conservative Dentistry, School of Dentistry, Kyung Hee University, 1 Hoe-gi-dong, Dongdaemoon-gu, Seoul, 02447 Korea; 2 Department of Periodontics, Seoul National University Dental Hospital, 101 Daehakno, Jongno-gu, Seoul, 03080 Korea; 3 Department of Conservative Dentistry, Kyung Hee University Dental Hospital, 1 Hoe-gi-dong, Dongdaemoon-gu, Seoul, 02447 Korea; 4 Department of Oral Science and Translational Research, College of Dental Medicine, Nova Southeastern University, 3200 S. University Dr., Fort Lauderdale, FL 33328 USA; 5 Department of Conservative Dentistry and Dental Research Institute, School of Dentistry, Seoul National University, 101 Daehakno, Jongno-gu, Seoul, 03080 Korea

**Keywords:** Light curing unit, Flexural strength, Micro-shear bond strength, Degree of conversion, Irradiation distance

## Abstract

**Background:**

The aim of this study was to evaluate the influence of increased irradiation distance on the flexural strength (FS), dentin micro-shear bond strength (μSBS), and the degree of conversion (DC) of bulk-fill flowable, conventional flowable, and packable resin composites.

**Methods:**

The resin composites tested were Surefil® SDR™ (SDR), Filtek Z350 XT Flowable Restorative A2 shade (Z3F), and Filtek Z350 XT Universal Restorative A2 shade (Z3P). Specimens were cured at four irradiation distances (0, 2, 4, and 8 mm) with an Elipar DeepCure-S LED curing light for 20 s. FS tests were performed (*n* = 15) using bar-shaped specimens (8 mm × 2 mm × 2 mm) of the resin composites. μSBS tests were performed on the occlusal surfaces of extracted third molars from humans that were ground to expose dentin (*n* = 15). DC was measured by using Raman spectroscopy on the top and bottom surfaces of disk specimens (2-mm thick) (*n* = 3). To further investigate whether extended irradiation times could compensate for reduced irradiance, additional Z3P specimens were prepared, which were light-cured at 8-mm distances for 40 and 60 s and subjected to FS tests, μSBS tests, and Raman spectroscopy. Both two-way and one-way ANOVA were used for statistical analyses.

**Results:**

Both FS and DC of Z3P specimens cured at an 8-mm distance were significantly lower than those cured at shorter distances (*p* < 0.05), whereas the FS and DC of the Z3F and SDR specimens were not significantly influenced by increasing distances. The μSBSs of the three types of resin composites reduced with increasing irradiation distances. The FS, μSBS, and DC of the Z3P specimen light-cured at 8 mm for 40 s were comparable to those of the Z3P specimen cured at 0 mm for 20 s.

**Conclusions:**

Increasing the irradiation distance to 8 mm can have a deleterious influence on mechanical performances, including the FS, DC, and dentin μSBS, of the resin composites polymerized with high-irradiance light curing units.

## Background

Light-cured resin composites have become the preferred materials to restore the enamel and dentin involved in dental caries, fractures, and noncarious cervical lesions; to use in endodontic procedures; and to correct the unesthetic colors and shapes of several etiologies [1]. These resin composites have excellent esthetic and mechanical properties because they have a tooth-like color and are similar or superior in strength to dentin or enamel [1, 2]. The optimal conversion of monomers into polymers initiated by light curing is essential to provide an adequate clinical performance to resin composites [3, 4]. Inadequate polymerization of resin composites can lead to the elution of monomers and deterioration of their mechanical properties, consequently causing secondary caries or fractures in restorations [5, 6]. An intimate proximity between the tip of a light curing unit (LCU) and a resin composite is required for achieving the best polymerization of the resin composite. However, the tip of an LCU should be placed away from the resin composite’s surface in some unavoidable cases. The curing distance between the LCU’s tip and the resin composite’s surface can exceed 8 mm in the restorations of deep proximal areas and core restorations involving root canal–treated teeth [7].

Previous studies have reported that an increased irradiation distance between the LCU’s tip and resin composites decreased the polymerization efficiency; therefore, a compensatory increase in irradiation time was recommended for achieving sufficient polymerization [7–11]. However, most of these previous studies were performed using conventional LCUs, which have much lower irradiance intensities than the LCUs currently in use [7–10]. Recently, LCUs have been developed to have wide wavelengths using multi-wave LED chips and an increased irradiance intensity of 1000 mw/cm^2^ or more. This increased irradiance intensity is much higher than 400 mW/cm^2^, the minimum irradiance of LCUs needed to polymerize resin composites [12, 13]. In addition, resin composites have been developed with new combinatorial compositions by introducing new photoinitiators, monomers, and fillers. These new types of resin composites, such as bulk-fill resin composites, are now being used in clinics [4, 14, 15]. The use of newly developed LCUs and resin composites has the potential to change the influence of irradiation distance on the polymerization efficiency of resin composites [4, 14, 15]. In fact, in studies using a high-irradiance LCU, contradictory results have been reported on the effect of increased irradiation distance on the mechanical performances of resin composites [15–20]. Therefore, additional studies to verify the effect of irradiation distance on the mechanical properties of resin composites are needed.

Although it is difficult for laboratory experiments to demonstrate the clinical performance of resin composites, several physical properties of resin composites are required to yield an acceptable performance in clinical conditions where close light curing is impossible [21–23]. Among various tests determining the physical properties of resin composites, the flexural strength (FS) test has been considered as a typical test to evaluate the complex mechanical behaviors and polymerization efficiency of resin composites in various load-bearing clinical conditions [24]. In addition, the degree of conversion (DC) is regarded as a critical parameter for determining the physical and biological properties of resin composites [24]. Moreover, increased irradiation distances can affect resin composites’ adhesion to dentin as well as the mechanical properties of the resin composite itself [11]. Low bond strength in deep proximal preparations and pulpal walls for core restorations may cause a poor performance in the reinforcement of a weakened tooth structure, thus influencing the longevity of resin composites [11]. Therefore, measurements of dentin bond strength should also be considered when determining the clinical performance of resin composites [11, 25]. To the best of our knowledge, few studies have simultaneously investigated the effect of irradiation distance on the FS, DC, and dentin bond strength of resin composites when curing with high-irradiance LCUs.

This study aimed to evaluate the influence of increased irradiation distance on the FS, DC, and dentin bond strength of bulk-fill flowable, conventional flowable, and packable resin composites using high-irradiance LCUs. The null hypotheses of this study were that there would be no significant differences in the FS, DC, and dentin bond strength of resin composites with changing irradiation distance and an increased irradiation time would not significantly change the FS, DC, dentin bond strength of resin composites cured at a long irradiation distance of 8 mm.

## Materials and methods

### Materials and experimental design

Three commercially available resin composites were used in this study: Surefil® SDR™ (SDR; Dentsply DeTrey GmbH, Konstanz, Germany) for the bulk-fill flowable resin composite, Filtek Z350 XT Flowable Restorative (Z3F; 3 M ESPE, St. Paul, MN, USA) A2 shade for the conventional flowable resin composite, and Filtek Z350 XT Universal Restorative (Z3P; 3 M ESPE) body A2 shade for the packable resin composite (Table 1). We used an LED LCU (Elipar DeepCure-S, 3 M ESPE; tip diameter = 10 mm) throughout this study. The intensity of the LCU was monitored using the built-in light intensity meter on the charging base.

**Table 1:** Materials used in this study

Materials (Code)	Manufacturer (Lot No.)	Composition	Filler loading (%)	Shade
Filtek Z350 XT Universal restorative (Z3P)	3 M ESPE, St. Paul, MN, USA (NA95885)	Bis-GMA, bis-EMA, UDMA, TEGDMA, 20-nm silica, 4–11-nm zirconia filler, Aggregated zirconia/silica cluster filler (cluster particle size of 0.6–10 µm)	63.3 vol./78.5 wt	Body A2
Filtek Z350 XT flowable (Z3F)	3 M ESPE, St. Paul, MN, USA (NA47040)	Bis-GMA, bis-EMA,TEGDMA, 0.1–5-µm ytterbium trifluoride, silica (20 and 75 nm), aggregated zirconia/silica cluster filler (cluster particle size of 0.6–10 µm)	46 vol./65 wt	A2
Surefil SDR flow (SDR)	Dentsply DeTrey GmbH, Konstanz, Germany (2,002,000,121)	Modified UDMA, TEGDMA, EBPDMA, barium-alumino-fluoro-borosilicate, glass, strontium-alumino-fluoro-silicate glass	44 vol./64 wt	Universal
Scotchbond Universal Etchant	3 M ESPE (6,697,834)	32% phosphoric acid	NA	NA
Adper Single Bond 2	3 M ESPE (NC05794)	Bis-GMA, HEMA, dimethacrylates, polyalkenoic acid copolymer, initiators, water, ethanol, and silica nanofillers	NA	NA

*NA* Not applicable

**Fig. 1:**
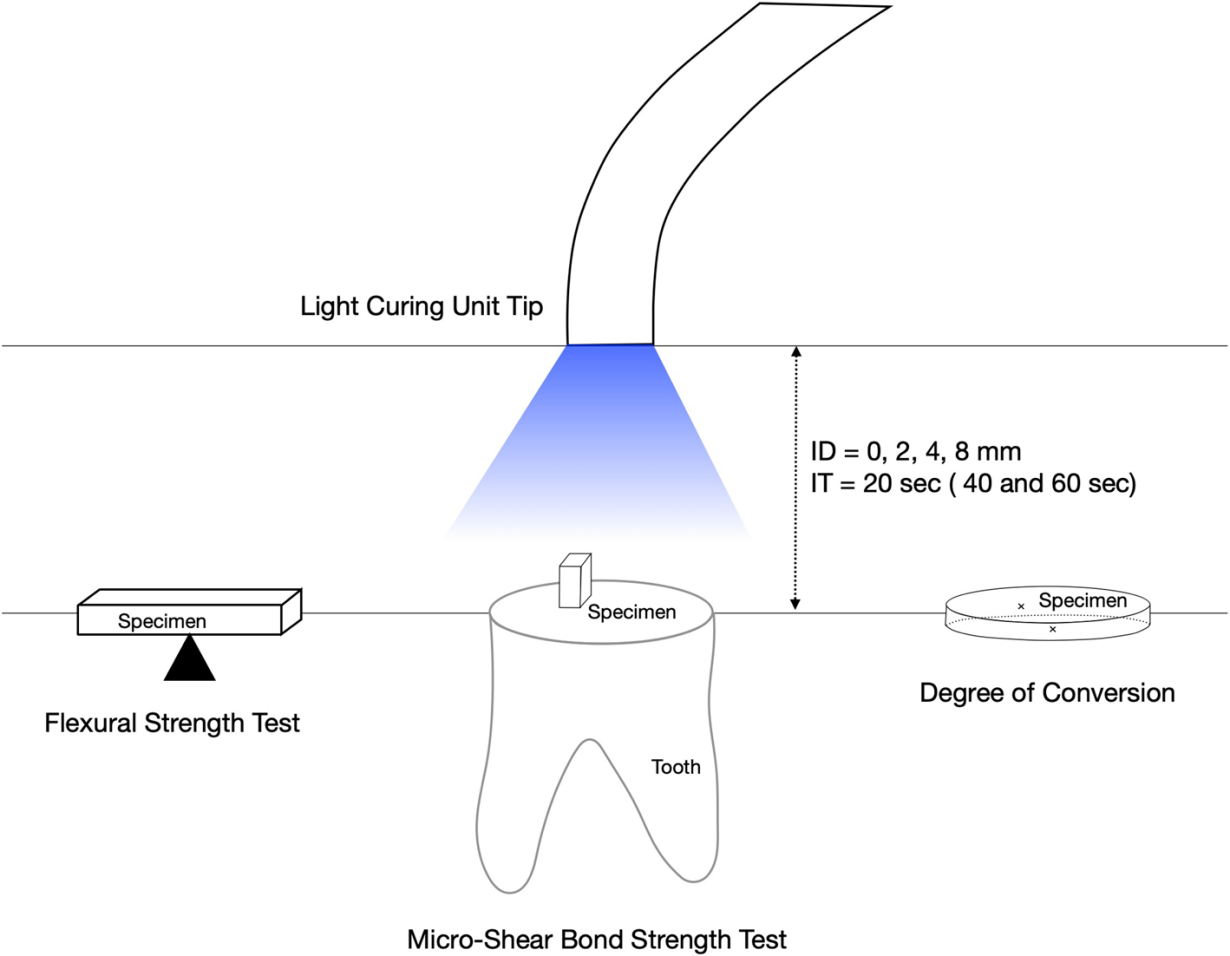
Experimental illustration of the study. (ID: irradiation distance; IT: irradiation time)

The experiments were designed to measure the FS, DC, and dentin bond strength of bulk-fill flowable, conventional flowable, and packable resin composites at different irradiation distances using the high-irradiance LCU. The scheme of the experimental design is shown in Fig. 1.

### Flexural strength test

Bar-shaped specimens were prepared using a custom mold (8 mm × 2 mm × 2 mm) made of vinyl polysiloxane impression material. The mold was filled with the resin composites, covered with polyester strip, and then, gently pressed using a glass slide to remove excess material. The prepared specimens were light-cured using the LED unit at four different irradiation distances (0, 2, 4, and 8 mm) from the specimens’ top surfaces (*n* = 15). For a distance of 0 mm, light curing was performed directly over the polyester strip. The position of the LED unit was standardized and centered over the specimen using a mechanical holder. The light curing was performed for 20 s. To evaluate whether extended curing times could compensate reduced irradiance at long curing distances, 30 Z3P specimens were light-cured at an 8-mm distance with light curing times of either 40 or 60 s (*n* = 15).

After light curing, all specimens were removed from the mold and stored in distilled water at 37 °C for 24 h before testing. During storage, the container holding the specimens was covered with aluminum foil to prevent the leakage of ambient light.

To evaluate the FS of the resin composites, the specimens were subjected to three-point bending tests using a universal testing machine (AGS-X STD, Shimadzu, Kyoto, Japan) at a crosshead speed of 0.5 mm/min until fracture. The load cell had a capacity of 2 kN, and the distance between the two supporting rods was 5 mm. The load was applied at the center of each specimen’s top surface using a third rod (2-mm diameter) until fracture was recorded, and the FS (in MPa) was calculated according to the following equation:

$$FS = 3Fl/2bd2$$

where *F* is the maximum load exerted on the sample (N), *l* is the distance between the two supports (mm), and *b* and *d* are the width and height of the specimen (mm), respectively.

### Micro-shear bond strength (μSBS) test

Extracted caries-free human third molars kept in 0.5% chloramine-T solution at 4 °C for less than 3 months were used. The study’s protocol involving the use of teeth extracted from humans was approved by the Institutional Review Board (KH-DT21005). The occlusal portion of the tooth was removed to expose its flat dentin surface using a high-speed diamond saw (IsoMet 5000, Buehler, Lake Bluff, IL, USA) under running water, followed by serial grinding with SiC abrasive papers with up to a #600 grit to obtain a standard smear layer. The occlusal dentin was etched with 32% phosphoric acid (Scotchbond Universal Etchant, 3 M ESPE) for 15 s and rinsed. After blot drying of the etched surface of the occlusal dentin, we applied two-step etch-and-rinse adhesives (Adper Single Bond 2, 3 M ESPE) to the specimens, followed by air drying and light curing using the LED unit for 10 s. To simulate the clinical cavity depth, the distances between the dentin and LCU were 1, 2, 4, and 8 mm in the 0, 2, 4, and 8-mm irradiance distance groups, respectively. A cylinder-shaped resin composite prepared by filling a polyethylene tube (0.8-mm diameter and 2-mm high) with the resin composite was attached to the adhesive-applied dentin surface. The specimens were light-cured using the LED unit for 20 s at four different irradiation distances (0, 2, 4, and 8 mm) from the specimens’ top surface (*n* = 15). To investigate the effect of extended irradiation times on the μSBS, an additional 30 Z3P specimens were prepared and light-cured at an 8-mm distance with curing times of either 40 or 60 s (*n* = 15). The samples were immersed in distilled water at 37 °C for 24 h before testing. During storage, the container filled with the samples was covered with aluminum foil to prevent the leakage of ambient light.

The bonded-tooth samples were mounted to the universal testing machine with the force vector parallel to the bonded surface. A stainless steel orthodontic wire with a 0.2-mm diameter was used to apply the shear force to the bonding interface of the sample. The wire was looped around the resin composite cylinder as close as possible to the bonding interface and connected to the arm of the universal testing machine. The tensile force was applied at a crosshead speed of 0.5 mm/min until failure occurred. The fracture load was recorded and divided by the area of the bonded surface (0.4 mm × 0.4 mm × π) to calculate the μSBS.

Following the μSBS test, the specimens’ surfaces were examined using a stereomicroscope at × 50 magnification to determine the failure mode: adhesive failure at the interface of the dentin and resin composite; cohesive failure in the resin composite; cohesive failure in the dentin; or a mixed failure (partial cohesive and adhesive failures).

### Degree of conversion

Raman spectra were recorded to calculate the DC of the resin composites according to the irradiation distance. We prepared three samples of each resin composite for four different irradiation distances (0, 2, 4, and 8 mm). To investigate the effect of extended irradiation time on the DC of the samples irradiated at long distances, additional Z3P specimens were light-cured at 8 mm and for 40 and 60 s (*n* = 3). A custom-made silicone mold with a cylindrical window (5-mm diameter and 2-mm high) was placed on a glass slide for sample preparation. The resin composite was inserted into the cylinder in a single increment, and a polyethylene strip was placed on top of the unpolymerized composite. A glass slide was placed on top of the polyethylene strip, and a 500-g load was applied for 30 s on the silicon mold. After the top glass slide was removed, light curing was performed with the above-noted respective light curing distances. Raman spectra were collected from the top and bottom surfaces of the prepared resin composite disk samples after 24-h storage in a dark container at 37℃ with 100% relative humidity. Raman spectroscopy measurements were performed using DXR2xi (ThermoFisher Scientific, Waltham, MA, USA). The excitation source was a diode laser with a 532-nm wavelength, a laser power of 10 mW, and a resolution of 2 cm^−1^. The acquisition range was between 1400 and 1700 cm^−1^, the exposure time was 0.1 s, and there were 1000 accumulations.

The DC (%) of the Z3F and Z3P were calculated by comparing the relative changes in the peak heights of the spectral bands at 1640 cm^−1^ (aliphatic C = C) and 1610 cm^−1^ (aromatic C = C) before and after light curing, respectively. The DC of the SDR was calculated by comparing the relative changes in the peak heights of the spectral bands at 1640 cm^−1^ (aliphatic C = C) and 1600 cm^−1^ (C-H stretching mode) before and after light curing using the following equation:

$$\mathrm{DC }({\%}) = 100 \times [1 - (R\mathrm{cured}/R\mathrm{uncured})$$

where *R* = peak height at 1640 cm^−1^/peak height at 1610 cm^−1^ for Z3F and Z3P, and *R* = peak height at 1640 cm^−1^/peak height at 1600 cm^−1^ for SDR.

### Statistical analysis

Normal distribution of data and homogeneity of variances were verified using Shapiro–Wilk and Levene’s tests, respectively. Data were analyzed by one-way and two-way ANOVA (irradiation distance vs. composite resin), and Tukey’s tests were performed for pair-wise comparisons (α = 0.05). All analyses were performed using SPSS for Windows, v23.0 (IBM Corp., Chicago, IL, USA).

## Results

### Flexural strength test

According to the two-way ANOVA, “irradiation distance” and “resin composite” were significant factors in determining the FS (*p* < 0.001 and *p* = 0.001, respectively), and the interaction of these two factors was significant (*p* < 0.001). All the composite groups revealed no statistically significant difference in the FS for all the irradiation distances used in this study, except for the Z3P, which showed a significantly lower FS at an 8-mm irradiation distance than at shorter irradiation distances (0, 2, and 4 mm) (*p* < 0.05) (Table 2). The mean FS of the Z3P was significantly higher than the mean FSs of the Z3F and SDR at irradiation distances of 0, 2, and 4 mm (*p* < 0.05), whereas there were no significant differences between the three resin composites at an 8-mm irradiation distance (*p* > 0.05) (Table 2). The SDR exhibited the lowest FSs at 0, 2, and 4-mm irradiation distances (*p* < 0.05) but did not showed a significant difference with the Z3F at a 0-mm irradiation distance (*p* > 0.05).

**Table 2:** Mean flexural strength (FS; MPa) of resin composites according to the irradiation distance

Resin composite	Irradiation distance (mm)			
	0	2	4	8
Z3P	166.27 ± 21.11^a,A^	167.20 ± 9.72^a,A^	162.88 ± 10.19^a,A^	142.29 ± 16.42^b,A^
Z3F	141.64 ± 9.75^a,B^	140.08 ± 9.94^a,B^	140.77 ± 9.88^a,B^	132.80 ± 6.9^a,A^
SDR	133.95 ± 13.85^a,B^	129.82 ± 12.89^a,C^	127.69 ± 11.16^a,C^	135.83 ± 8.16^a,A^

Different superscript lowercase letters in the same row indicate a significant difference according to the irradiation distance (*p* < 0.05). Different superscript uppercase letters in the same column indicate a statistically significant difference between the composite resins at the same irradiation distance (*p* < 0.05)

### Micro-shear bond strength test

**Fig. 2:**
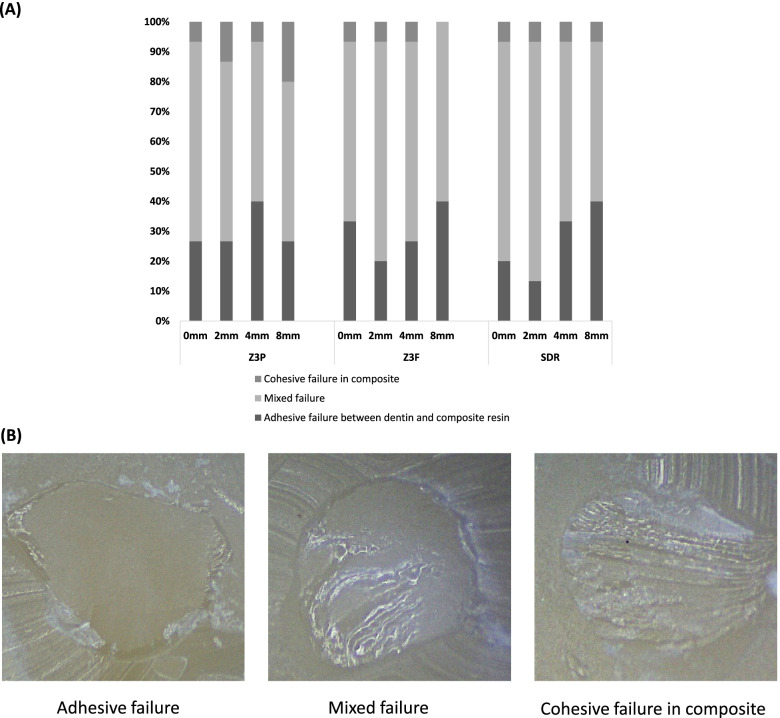
**A** Failure mode distribution and **B** representative optical images of each failure pattern (× 50 magnification) after micro-shear bond strength tests

According to the two-way ANOVA, the irradiation distance was a significant factor in determining μSBS (*p* < 0.001), whereas “resin composite” and its interaction with “irradiation distance” were not significant (*p* = 0.064 and *p* = 0.47, respectively). All the resin composite groups showed no statistically significant differences in μSBSs at 0-, 2-, and 4-mm irradiation distances (*p* > 0.05). An 8-mm irradiation distance led to statistically significant lower μSBSs than at shorter irradiation distances (0, 2, and 4 mm) (*p* < 0.05), except for the Z3F, for which 4- and 8-mm distances revealed no statistically significant differences (*p* > 0.05) (Table 3). The three composite resins used in this study had no significant differences in μSBSs for each irradiation distance (*p* > 0.05) (Table 3). The distribution of the failure mode was comparable according to the irradiation distance or resin composite, and the mixed failure pattern was observed to be dominant (Fig. 2). The mixed failure pattern was 63.3%, the adhesive failure pattern between the dentin and resin composite was 28.9%, and the cohesive failure pattern in the resin composite was 7.8%.

**Table 3:** Mean micro-shear bond strength (μSBS; MPa) of resin composites according to the irradiation distance

Resin composite	Irradiation distance (mm)			
	0	2	4	8
Z3P	29.95 ± 4.63^a,A^	29.81 ± 4.32^a,A^	26.51 ± 3.69^a,A^	20.93 ± 3.93^b,A^
Z3F	28.37 ± 5.7^a,A^	28.1 ± 4.38^a,A^	24.39 ± 4.73^a,b,A^	21.64 ± 2.67^b,A^
SDR	26.68 ± 4.91^a,A^	26.11 ± 4.69^a,A^	25.36 ± 4.91^a,A^	21.44 ± 4.0^b,A^

Different superscript lowercase letters in the same row indicate a significant difference according to the irradiation distance (*p* < 0.05). Different superscript uppercase letters in the same column indicate a statistically significant difference between composite resins at the same irradiation distance (*p* < 0.05)

### Degree of conversion

**Fig. 3:**
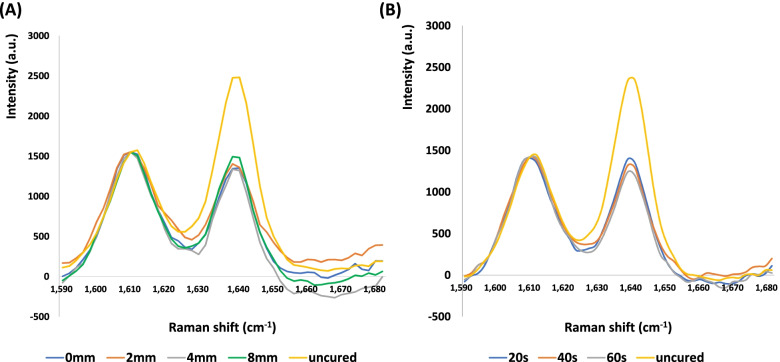
Representative Raman spectra of the Z3P in the spectral region of 1590–1680 cm^−1^. **A** Raman spectra of the Z3P according to the irradiation distance with a constant irradiation time of 20 s, **B** Raman spectra of the Z3P according to the irradiation time with an irradiation distance of 8 mm

The two-way ANOVA showed that “irradiation distance” and “resin composite” significantly influenced the DC (*p* < 0.05). The mean DC of the Z3P collected on the top and bottom surfaces tended to decrease with increasing irradiation distance (Table 4). For the top surface, the mean DC of the Z3P at an irradiation distance of 4 (45.57%) and 8 mm (44.22%) were lower than at 0 (50.52%) and 2 mm (50.88%) (*p* < 0.05). For the bottom surface, the mean DC of the Z3P at an irradiation distance of 8 mm (38.63%) was significantly lower than that at 0 (47.13%) and 2 mm (45.15%) (*p* < 0.05) (Table 4). At each irradiation distance, the SDR showed the highest DC (*p* < 0.05), and the Z3P and Z3F had no significant differences (*p* > 0.05) for the top and bottom surfaces. The ratio of the DC collected on the bottom surface to the top surface was above 0.8 regardless of the type of the resin composite and irradiation distance. Figure 3A shows the representative Raman spectra of the Z3P at different irradiation distances (0, 2, 4, and 8 mm). The height of the peak at 1640 cm^−1^ gradually increased as the irradiation distance increased up to 8 mm.

**Table 4:** Degree of conversion (DC; %) of resin composites according to the irradiation distance

Surface	Resin composite	Irradiation distance (mm)			
		0	2	4	8
Top	Z3P	50.52 ± 1.29^a,A^	50.88 ± 2.21^a,A^	45.57 ± 0.43^b,A^	44.22 ± 1.41^b,A^
	Z3F	46.91 ± 1.47^a,A^	46.52 ± 1.69^a,A^	46.13 ± 2.74^a,A^	43.47 ± 2.29^a,A^
	SDR	64.10 ± 5.18^a,B^	65.36 ± 4.49^a,B^	63.44 ± 5.86^a,B^	61.24 ± 3.64^a,B^
Bottom	Z3P	47.13 ± 2.55^a,A^	45.15 ± 2.46^a,A^	42.79 ± 0.99^a,b,A^	38.63 ± 0.86^b,A^
	Z3F	44.97 ± 1.63^a,A^	45.13 ± 4.66^a,A^	43.34 ± 0.56^a,A^	41.35 ± 2.51^a,A^
	SDR	61.16 ± 4.86^a,B^	59.83 ± 3.34^a,B^	61.23 ± 2.41^a,B^	57.28 ± 3.44^a,B^

Different superscript lowercase letters in the same row indicate a significant difference according to the irradiation distance (*p* < 0.05). Different superscript uppercase letters in the same column indicate a statistically significant difference between composite resins at the same irradiation distance on either the top surface or bottom surface (*p* < 0.05)

### Effect of extended irradiation times at an 8-mm irradiation distance for the Z3P specimen on the FS, μSBS, and DC

**Fig. 4:**
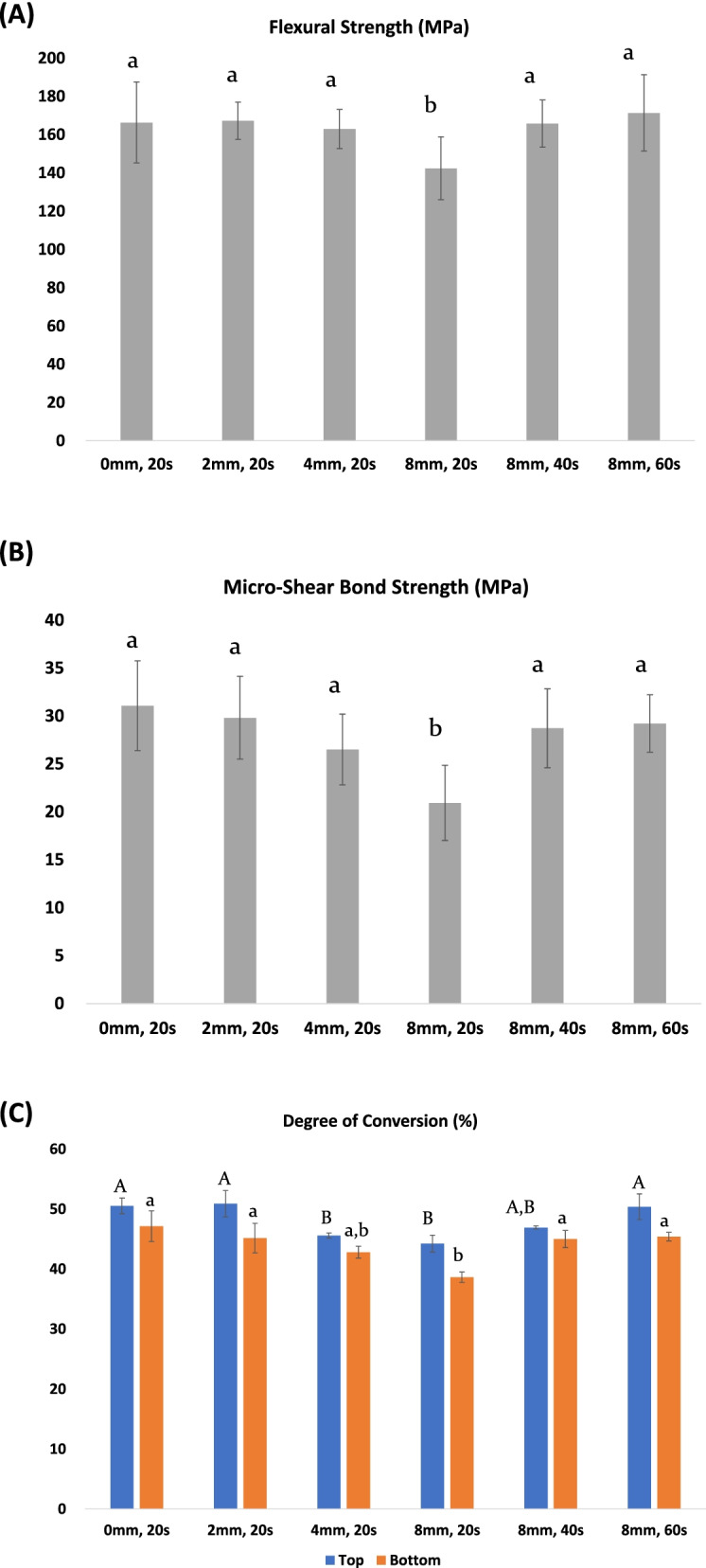
**A** Flexural strength, **B** micro-shear bond strength, and **C** degree of conversion of the Z3P with different irradiation distances and times. Different lowercase letters indicate significant differences between groups. **C** Different uppercase and lowercase letters indicate significant differences in the degree of conversion between groups collected on the top and bottom surface, respectively

Since the Z3P showed progressive deterioration in the FS, μSBS, and DC, it was additionally tested at an irradiation distance of 8 mm to determine whether extended irradiation times attenuate the effect of reduced irradiation. The peak height at 1640 cm^−1^ showed a gradually decreasing tendency with increasing irradiation times up to 60 s (Fig. 3B). An irradiation time of 40 s at 8 mm led to comparable FS, μSBS, and DC values with an irradiation time of 20 s at 0 mm (*p* > 0.05) Increasing the irradiation time to 60 s caused no significant differences in the FS, μSBS, and DC compared with an irradiation time of 40 s (*p* > 0.05) (Fig. 4).

## Discussion

We investigated whether the FS, DC, and dentin bond strength of the resin composites were affected by the irradiation distance and irradiation time in cases using the high-irradiance LED unit. The FS, μSBS, and DC of Z3P were significantly reduced with an irradiation distance of 8 mm compared with shorter distances (0, 2, and 4 mm). The μSBSs of the Z3F and SDR at an 8-mm irradiation distance were significantly lower than the μSBSs of the Z3F and SDR at shorter irradiation distances, whereas the FS and DC of the Z3F and SDR remain unaffected with increased irradiation distance. According to these overall results, our null hypothesis that there would be no significant differences in the DC, FS, and dentin bond strength with increasing irradiation distance was rejected. Our results showed that increasing irradiation distance could significantly affect the FS, DC, and dentin bond strength of the resin composites even with the high-irradiance LCU.

Some studies reported that high intensity LED LCUs did not cause reduced microhardness of resin composite with increased irradiation distance [16, 20]. On the contrary, the FS, μSBS, and DC of the Z3P resin composite were all negatively affected by increased irradiation distances as observed in the present study, which used the LED LCU with a high intensity of over 1,000 mW/cm^2^ [26]. It is clear from our study that the type of resin composite is a factor affecting the composite’s physical properties according to the irradiation distance. As we observed, the high intensity LED LCU we used did not lead to significant differences in the FS and DC between the irradiation distances of 0 and 8 mm in more translucent resin composites (Z3F and SDR) [27]. For the Z3P, its relatively higher opacity compared with the Z3F and SDR combined with the reduction of light intensity with increased irradiation distance might have hindered the light’s ability to reach the bottom of the resin composite, significantly reducing the FS, μSBS, and DC at an irradiation distance of 8 mm [28–30]. The effect of the increased irradiation distance on the μSBSs of the resin composites to dentin has been not been extensively investigated to date. The μSBSs of all three composite resins significantly reduced with an increased irradiation distance to 8 mm (Table 3). The bond strength of a resin composite to a tooth is determined not only by the performance of the adhesive but also by the physical properties of the resin composite applied on the bonding agent [11, 31]. Since both the adhesives and resin composites experienced reduced irradiation (resulting from an 8-mm irradiation distance) separately during the experiment, the combined effect of their reduced polymerization might have resulted in the decrease in the μSBS of all three of the study’s resin composites.

We also investigated whether increased irradiation time (40 s and 60 s) could compensate the Z3P’s reduced FS, DC, and μSBS at an irradiation distance of 8 mm. Increasing the irradiation time to 40 s was able to compensate the reduction in FS, DC, and μSBS (Fig. 4). However, further extending the irradiation time to 60 s did not provide any additional beneficial effect compared with an irradiation time of 40 s. Previous studies have shown that in cases of low irradiances resulting from increased irradiation distances two to three times longer irradiation times are required to compensate the reduced strength and hardness of resin composites [10, 32]. The results of the present study suggested that even with the high-powered LED LCUs commonly used today, it is advisable to light-cure the resin composites longer than the manufacturer’s recommended time if the light guide tip is more than 8 mm away from the cavity floor.

The FS of the Z3P was higher than that of the Z3F and SDR, except for the case of 8-mm irradiation distance. The Z3P, a conventional packable resin composite, has a higher filler composition than conventional flowable (Z3F) or bulk-fill flowable (SDR) resin composites (Table 1). More filler loading to a resin composite generally results in increased mechanical properties, such as elastic modulus, FS, and hardness [33, 34]. The Z3F and SDR have similar amounts of filler loading, except for the SDR at an 8-mm irradiation distance, the Z3F at all other irradiation distances showed a higher FS than the SDR. The components of monomers and fillers are considerably different in the Z3F and SDR. The Z3F has bis-GMA in the monomer composition and contains aggregated and nonagglomerated silica fillers. The SDR does not contain bis-GMA nor aggregated filler; instead, it contains modified UDMA and a large amount of glass filler. The compositional difference between the two resin composites may have resulted in different FSs. The DC of the SDR was the highest regardless of the irradiation distance in this study. The SDR’s UDMA-based monomer composition and high proportion of glass fillers as well as its proprietary photo-initiator system may have contributed to its higher DC than that of the other resin composites [35–37]. The SDR’s larger filler particle size compared with the Z3F and Z3P may also have led to its higher light transmission, thereby enhancing the polymerization reaction [38].

The gingival wall in a proximal box or the pulpal wall in a root canal–treated tooth can be as far as 8 mm from a light guide tip during resin composite restoration. The FS of the Z3P measured at 0, 2, and 4 mm were comparable (166.27, 167.20, and 162.88 MPa, respectively). The Z3P’s FS decreased to 142.29 MPa at an 8-mm distance, which is approximately 85% of the FS observed at shorter irradiation distances. The decrease in the μSBS of the Z3P at 8-mm irradiation distance was approximately 70%–80% of the μSBSs at shorter distances. Despite these reduced values, it is difficult to predict to what extent these reduced FS and μSBS values may result in compromised clinical outcomes. ISO 4049 stipulates that a specimen should be fabricated with dimensions of 2 mm × 2 mm × 25 mm for the FS test. However, three overlapping irradiation sequences are required to fabricate a specimen with a length of 25 mm due to the smaller diameter of the LCU (10 mm), resulting in nonuniform polymerization [39]. Therefore, we prepared specimens with dimensions of 2 mm × 2 mm × 8 mm for the FS test in this study, enabling single light curing.

Another limitation of this study was that we only used one type of LCU. Since the LCUs of every manufacturer have different spectral emissions and the amount of decreased irradiance with distance varies among different LCUs [14], additional studies are needed to examine the various types of LCUs commonly used in clinics. In addition, considering the fact that the SDR, as a bulk-fill composite resin, can generally be layered thicker than 2 mm, the combined effect of the applied resin composite’s thickness and the irradiation distance should be investigated in future studies.

## Conclusions

Increasing the irradiation distance to 8 mm can have a deleterious effect on the mechanical properties, including the FS, DC, and dentin μSBS, of the resin composites polymerized with the high-irradiance LCU. Therefore, even when using the high-irradiance LCU, compensatory extended irradiation times beyond the manufacturer’s recommendation are suggested in cases involving the restoration of deep preparations, such as deep proximal boxes or the pulpal walls of root canal–treated teeth.

## Data Availability

Please contact the corresponding author for data requests.
